# Effects of Ionizing Radiation on Flora Ten Years after the Fukushima Dai-ichi Disaster

**DOI:** 10.3390/plants11020222

**Published:** 2022-01-15

**Authors:** Gian Marco Ludovici, Andrea Chierici, Susana Oliveira de Souza, Francesco d’Errico, Alba Iannotti, Andrea Malizia

**Affiliations:** 1 Department of Industrial Engineering, University of Rome Tor Vergata, Via del Politecnico 1, 00133 Rome, Italy; gianmarco.ludovici@alumni.uniroma2.eu (G.M.L.); alba.iannotti@uniroma2.it (A.I.); 2 Department of Civil and Industrial Engineering, University of Pisa, Largo Lucio Lazzarino, 56122 Pisa, Italy; a.chierici@studenti.unipi.it (A.C.); francesco.derrico@ing.unipi.it (F.d.); 3 Physics Department, Federal University of Sergipe, UFS, Av. Marechal Rondon, s/n Jardim Rosa Elze, São Cristóvão SE 49100-000, Brazil; sosouza@academico.ufs.br; 4 Department of Biomedicine and Prevention, University of Rome Tor Vergata, Via di Motpellier 1, 00133 Rome, Italy

**Keywords:** ionizing radiation, radionuclides, Fukushima accident, higher plants, radio-resistance

## Abstract

The aim of this work is to analyze the effects of ionizing radiation and radionuclides (like ^137^Cs) in several higher plants located around the Fukushima Dai-ichi Nuclear Power Plant (FNPP), evaluating both their adaptive processes and evolution. After the FNPP accident in March 2011 much attention was focused to the biological consequences of ionizing radiation and radionuclides released in the area surrounding the nuclear plant. This unexpected mishap led to the emission of radionuclides in aerosol and gaseous forms from the power plant, which contaminated a large area, including wild forest, cities, farmlands, mountains, and the sea, causing serious problems. Large quantities of ^131^I, ^137^Cs, and ^134^Cs were detected in the fallout. People were evacuated but the flora continued to be affected by the radiation exposure and by the radioactive dusts’ fallout. The response of biota to FNPP irradiation was a complex interaction among radiation dose, dose rate, temporal and spatial variation, varying radiation sensitivities of the different plants’ species, and indirect effects from other events. The repeated ionizing radiations, acute or chronic, guarantee an adaptation of the plant species, demonstrating a radio-resistance. Consequently, ionizing radiation affects the genetic structure, especially during chronic irradiation, reducing genetic variability. This reduction is associated with the different susceptibility of plant species to chronic stress. This would confirm the adaptive theory associated with this phenomenon. The effects that ionizing radiation has on different life forms are examined in this review using the FNPP disaster as a case study focusing the attention ten years after the accident.

## 1. Introduction

Following the Fukushima Dai-ichi Nuclear Power Plant (FNPP) accident in March 2011, due to the Great Eastern Japan earthquake and tsunami, massive amounts of radioactive materials were released into the environment. Due to the direction of the wind, the great majority of these materials poured into the Pacific Ocean; however, some of them spilled over to coastal areas, causing soil contamination by radionuclides, mainly in Fukushima prefecture [1]. Among the radionuclides most deposited in the soil, ^137^Cs is the most dangerous as it has a relatively long half-life compared to other radioactive substances released by FNPP [2], this is way the authors have focused the attention on this radionuclide for this work. In addition, ^137^Cs contaminated soil binds strongly to clay and the migration rate of clay-bound ^137^Cs exhibits low mobility, less than 1 cm per year, suggesting that most of ^137^Cs is superficially distributed in the soil. ^137^Cs can emit γ-rays; hence, unusually high air dose rates continue over land areas [3]. In addition, although the number of radionuclides released in the coastal area decreased, they continued to diffuse from the FNPP through the aquifers. Consequently, all the flora and fauna present at the time of the accident received and continue to receive high doses of radiation from Fukushima. Therefore, the finding of adverse effects in wild organisms in the Fukushima area resulting from long-term, low-dose radiation exposure is of great concern [4]. Over the years, several investigations have tried to determine the levels of contamination with radioactive materials or to estimate the doses of radiation exposure in terrestrial organisms living around Fukushima. However, there are few studies on the impacts of environmental radiation on wild organisms. Furthermore, flora and wildlife are strongly influenced by human activities [4,5]. Following the incident, the Japanese government designated “Areas where residents are not allowed to live” and “Areas where residents are expected to have difficulty returning for a long time” near the FNPP which have higher annual radiation doses to 20 mSv. The result was a mass evacuation from these areas in the long term. While radiation levels in most of the evacuation zone are not considered extremely lethal to wildlife, land use change due to decontamination activities and the cessation of agricultural activities are believed to significantly affect flora, fauna, and ecosystems in these areas [6,7,8,9].

Specifically, we will discuss the general effects of ionizing radiation on higher plants focusing in detail on the morphological changes in Japanese conifers (mainly Japanese fir and Japanese red pine) ten years after the accident and on the current situation. Finally, we will evaluate the effects from a medical point of view and the possible improvement of emergency management, in detail the health one.

## 2. The Effects of Ionizing Radiation on Plants

**Figure 1:**
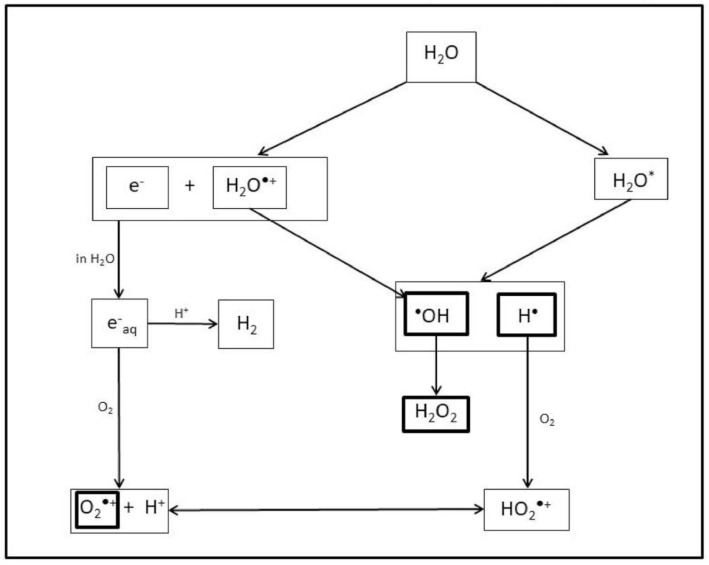
Primary (**^•^**OH, H**^•^**) and secondary (H_2_O_2_, O_2_**^•^**^−^) ROS involved in the oxidative stress produced by Ionizing Radiation (e_aq_^−^: solvated electron; H_2_O*: excited water molecule).

Ionizing radiation can deposit energy in a system and it is ubiquitous in the environment. Its source can be natural such as radioactive materials and cosmic rays or artificial, such as nuclear power plants [10]. Recently, there has been a strong interest in the health of organisms in radioactively contaminated sites such as those of Chernobyl and Fukushima Dai-ichi. Many wildlife species have surprised many scientists, but there are also reports of significant effects of chronic irradiation at relatively very low doses [11,12]. This contradiction has been observed at doses of environmental radioactivity and has yet to be fully understood. It is important to do this because ionizing radiation, although present in nature, can also derive from human activities. The exposure of a biological system to ionizing radiation activates a series of signals that start with the absorption of energy and continue in biological lesions [13]. There are two types of interactions, direct and indirect. In direct, the energy of the radiation is deposited directly in the targets. In indirect actions, on the other hand, the energy is first absorbed by an external medium, leading to the production of diffusible intermediates which subsequently attack the targets. The main target in both interactions of ionizing radiation is the H_2_O molecule, present in all organisms [11,14]. The primary reactions are excitation and ionization, which produce ionized water molecules (H_2_O^•+^) and the radicals H^•^ and ^•^OH (Figure 1) [11,14]. In addition, in a biological system this type of ionization is induced along the entire path of the radiation, triggering chain reactions, which produce secondary reactive oxygen species (ROS) because of H^•^ and e_aq_^−^ becoming trapped [15,16]. The most important ROS is H_2_O_2_; O_2_^−•^ is produced in low doses, based on molecular oxygen levels. The •OH radical can react very quickly with different types of macromolecules including lipids and proteins, but especially with Deoxyribonucleic Acid (DNA) [11,15,16]. However, depending on the dose, many of the resulting injuries can be readily mitigated and repaired. Radio-induced DNA damage is mainly caused by indirect effects and is considered the most important, although direct effects may contribute to the damage [17]. Consequently, based on the dose and radio-sensitivity of the species, both genomic and chromosomal aberrations are generated. Generally, DNA is a candidate to be the primary site of radiation damage, thus explaining the resulting radiation-induced mitotic arrest [17,18]. Furthermore, there are cellular mechanisms that allow damaged cells to repair the damage; however, errors in DNA repair naturally occur and this leads to aberrations and subsequent transmission to descent [19,20]. Thus, cell division in the meristem or germline responds drastically to ionizing radiation. It is difficult to compare current data on plant responses to ionizing radiation as the conditions of the models and parameters of past experiments are very different [21]. Therefore, the type of irradiation (acute or chronic), the dose rate or dose applied, the physiological parameters such as species, varieties, cultivars considered, the stage of development at the time of irradiation and the variations of the individual could be different between studies. The current inhomogeneity is further aggravated by the coexistence of different experimental data, data applied by the food industry and data relating to accidents [22,23]. Consequently, in the experimental field of plant radiation, doses can vary from a few Gy to several hundred Gy but can also reach the level of kGy. Furthermore, the dose range response strongly depends on the species studied. It is difficult to predict a standard response to ionizing radiation in plants even though promising standardization schemes are emerging [11,22,24].

The effects of ionizing radiation in higher plants are of interest to agriculture, ecology, health, and new space frontiers [11,12]. In general, four fundamental aspects of plant biology need to be considered as they provide a vital context for analyzing the effects of ionizing radiation [22]. The light reactions of photosynthesis start with photolysis of H_2_O; consequently, this process produces large quantities of ROS, the same products of H_2_O radiolysis that plants are generally able to block thanks to the large production of antioxidants [25]. It must be considered that in multicellular plants, cells divide into meristematic tissues, and they have quiescent centers with the same functionality as stem cells but are not identical to each other [11,22]. For example, they lack the apoptotic response of animal stem cells, mediated by the p53-oncoprotein. Meristems in plants are a biologically distinct product resulting from an independent evolution of multicellularity; the effects of ionizing radiation on them are not yet well known [22]. It is important to note that the meiotic divisions that produce the generation of gametophytes in the reproductive organs of plants are separated in each generation by many divisions of vegetative cells in the generation of sporophytes; in detail, the plants alternate between generations and no dedicated germ line [26]. Although tumors can occur in plant tissues, thanks to multiple controls on dividing plant cells and the low probability of metastasis, being organisms without a circulatory system, plants do not suffer the oncogenesis effects, typical of many animals [22]. Hence, plants are unlikely to have the same stochastic effects as ionizing radiation in animals, where in many cases they cause carcinogenesis [22]. Therefore, the current information on the effects of ionizing radiation on multicellular organisms is provided by the knowledge of the effects on organisms with lower antioxidant capacity than plants, which possess stem cells and germ lines without equivalent in plants and which suffer from stochastic effects that probably do not occur in the plant kingdom [11,22,27].

## 3. Fukushima Dai-Ichi Overview

**Figure 2:**
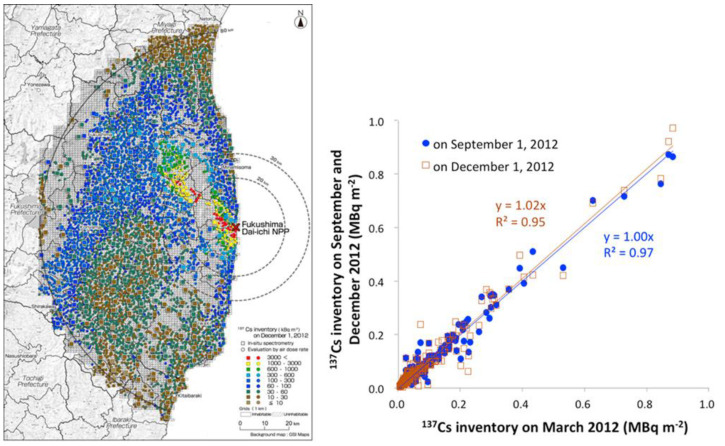
Radionuclides ground deposition from the FNPP Accident [32,33].

At the time of the accident, in the areas around FNPP, there were several warm temperate forests that have suffered radionuclides deposition (Figure 2). The forests of this area were and are mainly formed by coniferous plants such as Japanese red pine (*Pinus densiflora*), Japanese fir (*Abies firma*), Japanese cypress (*Chamaecyparis obtusa*), Japanese cedar (*Cryptomeria japonica*) and broad-leaved trees such as Konara oak (*Quercus serrata*) [8,28,29,30]. Several studies have analyzed these higher plants in the forests of the ex-evacuation zone. Precisely in this area, about eight months after the accident, in November 2011, a thorough investigation was conducted, but no chronic radiation injuries such as morphological anomalies or yellowing and fall of the leaves were observed [30,31,32,33]. These same observations also occurred in the forest most contaminated by the accident, about 3 km west of FNPP [28,29,30]. Contrariwise to the Chernobyl Nuclear Power Plant (CNPP) accident where radiation injury was observed relatively early, in the case of the FNPP accident, large-scale radiation damage did not occur in plants following sub-acute exposure [12,34,35,36].

However, to evaluate the effects of ionizing radiation on conifers in the ex-evacuation zone over the medium and long term, further studies have been performed [28,30]. In January 2015, assessments were conducted in the area around FNPP. Specifically, morphology was observed in the Japanese fir population present in the forest [29,30]. In detail, the observation was performed as follows: within the ex-evacuation zone, three distinct zones were examined, each with an area of approximately 1 km^2^. Each of the three zones contained between 100 and 200 young Japanese fir, ranging in height from 40 cm to 5 m. A population of Japanese fir, far away from the accident site, was observed as a control [30]. Significant morphological changes were observed, in young Japanese fir populations, primarily at sites in the ex-evacuation zone with a particularly high environmental dose rate compared with the population at the control site with a low environmental dose rate. In addition, it was found that the frequency was higher depending on the environmental dose rate at each site [30,37]. Generally, Japanese fir trees have a single main trunk while the trees that showed more morphological changes were characterized by branching defects caused by deletion of the main shoot. Once the branching defect was identified on a tree-by-tree basis, an increase in this anomaly was evident in studies performed between 2012 and 2013 compared to a control performed in 2010, before the accident [28,30]. The results obtained in past experiments with γ-irradiation and the example of the CNPP incident, therefore, suggest that higher plants such as Japanese fir and Japanese red pine possess high sensitivity to ionizing radiation suggesting that this type of radiation contributed greatly to the morphological changes found in coniferous forests around FNPP [22,28,29,30,34,35,38]. A further study, conducted between 2014 and 2016 at eight sites in Fukushima Prefecture, including the former evacuation zone, demonstrated a common morphological change in Japanese red pines, namely the disappearance of apical dominance [29]. As shown by different studies, it was clear that the rate of morphological changes was directly proportional to the dose of ionizing radiation to which the conifers had been exposed and obviously by how much dose they had absorbed; these changes began to occur four years after the first exposure [28,29,30]. These observed morphological changes were comparable to those found in Scot’s pine within the Chernobyl Exclusion Zone (CEZ-zone within 30 km of the nuclear power plant) after the CNPP accident. This additional data supports the hypothesis that ionizing radiation are the cause of the morphological changes [12,34,35,39,40]. Further investigation of coniferous forests within the FNPP ex-evacuation zone did not show the type of large-scale radiation damage in contrast to that observed after acute radiation exposure in the early stages after the CNPP accident [28,29,30]. In addition, close observation of coniferous forests around FNPP revealed that the most pronounced morphological change was in the shoots of Japanese fir and Japanese red pine, suggesting the effect of chronic exposure. However, it should be emphasized that these types of changes may be caused naturally by environmental factors and not due solely to ionizing radiation [29]. Therefore, to elucidate and explain the relationship between ionizing radiation and morphology change in trees, it is first necessary to evaluate very accurately the radiation dose absorbed by conifers and then to which environmental factors they are commonly exposed, to assess by which precise process morphological changes occurred [41]. To assess morphological changes in coniferous forests due to exposure to ionizing radiation, comparisons should be made with analytical data, provided using experimental facilities such as gamma fields [30]. Data obtained from irradiation experiments on Japanese fir and Japanese red pine trees indicate that radiation sensitivity changes first according to the species of conifer analyzed and then according to the type of injury such as growth failure, reproductive effects, and finally, death [42]. These types of irradiation experiments on specific types of conifers should be considered under conditions as similar as possible to their growth environment to assess whether similar morphological change is observed around FNPP [28,30]. To reproduce as closely as possible an experimental situation like that which occurred at the FNPP, it is necessary to estimate as accurately as possible the radiation dose to which the conifers were exposed in the area of interest [30,41,43]. Therefore, once the radiation dose in conifers in the area of interest has been estimated and subsequently compared with the irradiation dose near the plant that generates radiation (e.g., CNPP and FNPP) that induces morphological changes in trees, it will be possible to clarify the relationship between the incidence of morphological changes in conifers in the analyzed area and radiation exposure [30]. It is therefore clear that it is necessary to create a dose assessment model to reproduce as closely as possible the exposures that the tissues of conifers receive from the various sources of radiation in the environment. The real challenge is, therefore, to reconstruct the variation in the exposed dose of conifers ten years after the FNPP accident based on actual measurements of the concentration of radionuclides in the surrounding forests [28,29,30,42,43] (Table 1). The experimentation of the past years and the one that is continuing nowadays has also provided additional elements to understand the correct correlation between ionizing radiation and morphological changes of coniferous forests around FNPP [43]. Moreover, after ten years, the constant observation, monitoring and control of the area around FNPP is of fundamental importance to guess the direction that morphological and genetic changes of conifers will take in the long term.

## 4. Current and Future Perspectives: Management in the Medical Field

Research into the long-term effects on flora, fauna and human health following the FNPP incident is constantly evolving and proceeding relentlessly [7]. The CNPP and FNPP accidents provide unique opportunities on the investigation of radiological consequences and radiation effects on environment in a large scale that cannot be observed in the laboratory, not only for the health of higher plants but also for human health [32,44,45,46,47]. Prior to the Fukushima incident, few decision makers paid attention to the need to plan health investigations following a large-scale radiation scattering incident [47,48,49]. Following the FNPP incident, despite the serious initial difficulties, the basic concept of the Fukushima Health Management Survey (FHMS) was developed, which defined not only the health effects of ionizing radiation, but also other problems, such as example mental health and the consequences of long-term relocation [50,51]. To cope with this emergency, SHAMISEN (Nuclear Emergency Situations—Improvement of Medical and Health Surveillance) was founded in 2016 and its activities are still largely active today [50]. It is a project funded by the Open Project For European Radiation Research Area, that aims to develop recommendations for medical and health surveillance of populations affected by previous and future radioactive incidents based on lessons learned from past incidents, including the CNPP and FNPP incidents [50,52,53]. SHAMISEN recommendations state that: “The management of radiological incidents also raises important ethical questions. Although most radiation protection actions, including health surveillance, are aimed at reducing the impacts of exposure to ionizing radiation, most of them lead to with it a multitude of direct and indirect consequences that can have a great impact on the well-being of the affected populations. Ethical considerations are also important for the design and implementation of health surveillance and epidemiological studies” [50,52,53]. In conclusion, given the purpose and current activity of SHAMISEN, it is currently, proving to be a useful tool to better manage future large-scale incidents with dispersion of ionizing radiation [52,53]. Ensuring a precise, targeted, and fast intervention in the management of emergencies is the ultimate goal of decision makers. Learning from past incidents and implementing this knowledge can make a significant difference in terms of lives and costs in healthcare management [54].

## 5. Conclusions

The accumulation and uptake of radioactive ^137^Cs in higher plants was studied extensively in the early stages following the FNPP accident. In addition, phytoremediation (the use of higher plants to clean up soil contaminants) was considered early in the aftermath of the event. Contrary to what was expected, the conducted attempts proved to be a failure as the uptake of radioactive ^137^Cs from the soil by the higher plants was lower than expected since the radioactive ^137^Cs transfer factor, i.e., the ratio of ^137^Cs concentration in the plants to the ^137^Cs concentration in the soil, was less than one in the higher plants. Several studies have shown that young Japanese fir trees growing around FNPP exhibited a significant increase in morphological changes in contrast to other sites, which were not exposed to ionizing radiation. These conifers showed irregular branching at the main axis whorls, with elimination of the main shoot and its subsequent termination with the resulting bifurcation of the lateral shoots. Consequently, it was shown how the frequency of these anomalies corresponded to the environmental radiation dose rate at the observed sites, contributing to the hypothesis that radionuclide contamination generated the morphological changes in coniferous forests around FNPP. Interestingly, the frequency of major shoot deletions was significantly highlighted after the spring of 2012, peaking throughout 2013. The reason why the highest frequency of abnormalities was observed two years after the FNPP incident remains unanswered. These studies concluded that further evidence is needed to elucidate the processes of apical shoot clearance resulting from ionizing radiation at the cellular and/or tissue level in the observed Japanese fir trees; this is the reason for the current ongoing studies. Morphological abnormalities in higher plants resulting from high levels of ionizing radiation at Fukushima were also found in another conifer species. It was observed that deletion of apical dominance was also present in young Japanese red pine populations, whereas no morphological effect was detected in mature populations of the species. The observed anomaly is very similar to that found in young Scot’s pine (*Pinus sylvestris* L.) trees in the 30 km CEZ. As we discussed the probability of apical dominance elimination in Japanese red pine populations is increased with increasing absorbed doses, confirming the hypothesis that the anomalies found in Japanese red pine may be attributable primarily to external ionizing radiation. All morphological abnormalities occurred four years after the start of exposure, and approximately 50% of all abnormalities detected appeared two years after the first exposure. An interesting factor is that the appearance of the apical dominance deletion was temporary, and no new abnormalities were observed in the five-year-old whorl in conifers. These temporal patterns of morphological change are like the cases of Scots pine at Chernobyl and Japanese firs at Fukushima. These results indicate that the morphological abnormalities found in conifer species could be caused by internal rather than external exposure, because the external exposure dose to these trees peaked during the first year after exposure to ionizing radiation. In conclusion, surveys of conifers within the ex-evacuation zone of the FNPP accident did not show the type of large-scale radiation injuries unlike those observed after subacute exposure to ionizing radiation in the early stages after the CNPP accident. On the other hand, the fact that morphological changes were observed more frequently in Japanese fir and Japanese red pine shoots around the FNPP further suggests the influence of chronic exposure.

Ten years have passed since the FNPP accident, and still the large-scale effects are visible as well as the morphological changes on flora and fauna. It will have negative repercussions for many generations. This work wants to underline how is important take care of the lessons learned during nuclear events to continuously improve the emergency management plans and the capabilities to monitor the health conditions of all the people exposed. This approach is fundamental to reduce the risk factors and provide a better life expectancy.

## Figures and Tables

**Table 1:** Effect of ionizing radiation on the main conifers around the FNPP zone.

Species		Effects	Reference
Japanese fir	*Abies firma*	Irregular branching at the main axis, resulting termination of the main shoot or forking of lateral shoots.	[28,30]
Japanese red pine	*Pinus densiflora*	Increased cancellation of apical dominance with increased absorbed dose rate. Frequencies of cytogenetic abnormalities in the intercalary meristem of needles. Significant increase of some phytohormones, principally auxin (IAA) concentrations was observed.	[28,29,43]

## Data Availability

Not applicable.
